# A 7 Tesla Amygdalar-Hippocampal Shape Analysis of Lithium Response in Bipolar Disorder

**DOI:** 10.3389/fpsyt.2021.614010

**Published:** 2021-02-16

**Authors:** Thomas L. Athey, Can Ceritoglu, Daniel J. Tward, Kwame S. Kutten, J. Raymond DePaulo, Kara Glazer, Fernando S. Goes, John R. Kelsoe, Francis Mondimore, Caroline M. Nievergelt, Kelly Rootes-Murdy, Peter P. Zandi, J. Tilak Ratnanather, Pamela B. Mahon

**Affiliations:** ^1^Center for Imaging Science, Johns Hopkins University, Baltimore, MD, United States; ^2^Institute for Computational Medicine, Johns Hopkins University, Baltimore, MD, United States; ^3^Department of Biomedical Engineering, Johns Hopkins University, Baltimore, MD, United States; ^4^Department of Psychiatry and Behavioral Science, Johns Hopkins School of Medicine, Baltimore, MD, United States; ^5^Department of Occupational Therapy, Boston University, Boston, MA, United States; ^6^Department of Psychiatry, VA San Diego Healthcare System, La Jolla, CA, United States; ^7^Department of Psychiatry, University of California, San Diego, La Jolla, CA, United States; ^8^Department of Psychology, Georgia State University, Atlanta, GA, United States; ^9^Department of Mental Health, Johns Hopkins Bloomberg School of Public Health, Baltimore, MD, United States; ^10^Department of Psychiatry, Brigham & Women's Hospital, Boston, MA, United States; ^11^Department of Psychiatry, Harvard School of Medicine, Boston, MA, United States

**Keywords:** lithium, 7T MRI, shape analysis, amygdala, hippocampus, bipolar disorder

## Abstract

Research to discover clinically useful predictors of lithium response in patients with bipolar disorder has largely found them to be elusive. We demonstrate here that detailed neuroimaging may have the potential to fill this important gap in mood disorder therapeutics. Lithium treatment and bipolar disorder have both been shown to affect anatomy of the hippocampi and amygdalae but there is no consensus on the nature of their effects. We aimed to investigate structural surface anatomy changes in amygdala and hippocampus correlated with treatment response in bipolar disorder. Patients with bipolar disorder (*N* = 14) underwent lithium treatment, were classified by response status at acute and long-term time points, and scanned with 7 Tesla structural MRI. Large Deformation Diffeomorphic Metric Mapping was applied to detect local differences in hippocampal and amygdalar anatomy between lithium responders and non-responders. Anatomy was also compared to 21 healthy comparison participants. A patch of the ventral surface of the left hippocampus was found to be significantly atrophied in non-responders as compared to responders at the acute time point and was associated at a trend-level with long-term response status. We did not detect an association between response status and surface anatomy of the right hippocampus or amygdala. To the best of our knowledge, this is the first shape analysis of hippocampus and amygdala in bipolar disorder using 7 Tesla MRI. These results can inform future work investigating possible neuroimaging predictors of lithium response in bipolar disorder.

## 1. Introduction

Bipolar I disorder (BD) is characterized by a relapsing and remitting course and is common, affecting an estimated 1% of the population (1). Treatment of BD is complex, often involves polypharmacy, and it can take months or even years to find an effective treatment for an individual patient (2). Lithium is a common mood-stabilizing treatment that has been shown to significantly reduce risk of depressive or manic relapse (3, 4). However, only about 50% of patients with BD respond to lithium (5). Identification of reliable predictors of treatment response could greatly reduce illness burden and improve the lives of patients with BD (6–8).

A limited number of predictors of lithium response in BD have been identified, including clinical and genetic features (6–9). Clinical predictors of positive response include an illness pattern of manic episodes before depressive episodes and later age of onset of the disorder. However, no single clinical feature has been found to strongly predict lithium response (8). In terms of genetics, Genome Wide Association Studies (GWAS) have now identified genetic variation associated with lithium response, including single nucleotide polymorphisms (SNPs) located in a region containing genes for long non-coding RNAs that regulate gene expression and in the genes SESTD1 and in GADL1 (10–12). Additional suggestive associations of lithium response have been reported with SNPs located in the gene GRIA2 and with microRNAs (13, 14).

Another data modality that may predict treatment response is neuroimaging (7). Decreased bilateral volumes of hippocampi, amygdalae, and thalamus, and increased lateral ventricle volume have been shown in BD, along with altered function and connectivity in related cortico-limbic circuits (15–20). Lithium treatment in BD has been associated with larger volumes of structures such as the hippocampus and amygdala, although not consistently, as well as hypoactivation in subcortical structures typically found to be hyperactivated in BD (18, 21–24). Only a few previous studies have used neuroimaging to examine response to lithium treatment, with some identifying patterns of structure and function in cortico-limbic regions and circuits consistent with a normalizing effect of lithium (25–29). Most studies examining lithium effects on structural MRI have examined brain volumes, cortical thickness or surface area, reducing all morphological information to a single statistic (17, 21, 22, 30). Exploring more local effects may provide additional information and potentially help further elucidate lithium's neurobiological action. While a few studies have examined subcortical structure at a more detailed level, with some reporting localized differences in hippocampus including in CA1, CA2/3 and subiculum, such studies assessed structural changes related to lithium use and did not take into account differences in individual responses to lithium treatment (23, 31–34).

In this study, we combined a focus on response to lithium treatment with an examination of local structural effects, utilizing Large Deformation Diffeomorphic Metric Mapping (LDDMM) methods to identify local morphological differences between patients with BD who responded to lithium monotherapy treatment, those who did not respond, and healthy comparison participants (HC). LDDMM methods can quantify local morphological differences in brain structures and have been used previously to study patterns of atrophy in diseases such as Alzheimer's and Huntington's (35, 36). Our goal was to identify amygdalar and hippocampal shape correlates of lithium response in BD. This preliminary study could help identify brain features to be examined in future neuroimaging studies to identify predictors of lithium response in BD.

## 2. Materials and Methods

### 2.1. Participants

Participants with BD were recruited at the Johns Hopkins site of the Pharmacogenomics of Bipolar Disorder Study (PGBD), an eleven site prospective trial of lithium monotherapy in adult patients with BD (37). Diagnostic and Statistical Manual of Mental Disorders (DSM-IV) research diagnosis was made by a psychiatrist using the Diagnostic Interview for Genetic Studies (DIGS) (38). Participants with BD were included if they (i) met DSM-IV criteria for bipolar I disorder, (ii) were currently euthymic with Beck Depression Inventory (BDI) <19 and Clinician-Administered Rating Scale for Mania (CARS-M) <8 (39, 40) and (iii) were enrolled in the PGBD study [for inclusion and exclusion criteria of that study see (37)]. HC were recruited from the community using flyers, or from participants in previous research studies at Johns Hopkins who had given written permission to be re-contacted for future research, and were included if they had (i) no self-reported psychiatric history based on the Mini-International Neuropsychiatric Interview (MINI) (41), (ii) no self-reported family history of psychiatric disorder in any first-degree family member. All participants met the inclusion criteria of (i) 18–65 years old, (ii) right handed and were excluded by (i) alcohol or substance abuse or dependence during the past 6 months, (ii) dementia or mild cognitive impairment, (iii) contraindication to an MRI scan.

As part of the PGBD study, participants with BD were followed through a 16-week stabilization phase to stabilize mood and titrate off psychotropic medications other than lithium, followed by a 4-week observation phase. During the 4-week observation phase, subjects with a Clinical Global Impression-Severity (CGI-S) score of 3 or less (mildly ill) for at least 4 weeks were advanced to a maintenance phase. During maintenance participants were assessed every 2 months for up to 24 months. Determination of lithium response was according to the PGBD study (37). Non-response was defined by failure to remit over the stabilization phase and/or observation phase, or relapse during the maintenance phase. Relapse was defined by either (i) meeting criteria for mania and having a CGI-S score of 4 or greater (markedly ill), (ii) meeting criteria for a major depressive episode with a 4-week duration, (iii) meeting criteria for a mixed episode with a CGI-S score of 4 or greater, (iv) psychiatric hospitalization for a mood episode, or (v) if in the physician's judgment, the patient could not be managed on monotherapy and required a change in medication. Response was defined at two time points: “acute response” considering whether the patient remained well enough to advance to the maintenance phase, and “long-term response” considering up to 24 months of follow-up during the maintenance phase.

Participants with BD were consented and enrolled into the MRI study after beginning the PGBD study. Of the 25 participants (17 female, 8 male) who consented to participate in the MRI study, two were later excluded due a change in mood disorder diagnosis, one due to treatment non-compliance, and one was unable to complete the MRI scan. Participants were scheduled for MRI scanning after a clinical determination of acute response was made. A total of 7 participants were lost to follow-up prior to the MRI scan. Thus, a clinical determination of lithium response and a completed MRI scan were available for 14 participants with BD (9 acute responders, 5 acute non-responders). Two acute responders were determined to be long-term non-responders. Twenty-one HC were enrolled into the study and scanned.

### 2.2. Clinical Assessment

On the day of the MRI scan, all participants completed the Hopkins Adult Reading Test (42) as an indicator of Full Scale IQ, as well as the BDI and CARS-M to assess current dessive and manic symptoms, respectively. Possible dementia and mild cognitive impairment were assessed using the Mini Mental Status Exam (43) and Montreal Cognitive Assessment (44).

As some participants with BD had initiated treatment with lithium prior to entering the study, duration of lithium monotherapy at the time of the scan ranged from 2 months to 12 years. The mean dose of lithium in the participants was 1, 000±300 MG. At the time of the MRI, four participants who had exited the PGBD study had recently added an antipsychotic or antidepressant medication.

### 2.3. Image Acquisition and Segmentation

T1-weighted MP-RAGE brain scans (TR = 4.3 ms, TE = 1.92 ms, axial orientation, matrix = 225 × 288 × 288, resolution = 0.8 × 0.764 × 0.764 mm) were acquired on a Phillips 7.0-Tesla scanner (32 channel head coil) at Kennedy Krieger Institute (Baltimore, MD).

Binary segmentations in the population were obtained using the multi-atlas random orbit model (45). First, multi-atlases of segmented hippocampi, amygdalae and coarse regions were created from a subset of the population and then used to generate segmentations in the entire population. The initial segmentation and editing were manually performed using Seg3D (46), summarized here.

1. A contributor who was unblinded to the subjects' clinical features selected 5 subjects who were representative of the larger cohort with respect to sex, age, education, and diagnosis. These subjects are henceforth referred to as the atlas subjects. All following steps were performed by a contributor who was blinded to the subjects' clinical features.
2. Skull strip masks were constructed manually for the atlas subjects. This segmentation followed the dura mater around the cerebrum and cerebellum. The inferior most slice was inferior border of the cerebellum (47).
3. The atlas subjects were segmented for left and right hippocampus, and left and right amygdala according to the Mai atlas (48).
  The amygdalae were segmented primarily in the coronal plane, similar to (49). In anterior slices of the amygdalae, white matter defined the ventrolateral and ventromedial borders. The dorsomedial border was defined by the semilunar gyrus. The lateral border was defined by the striations between the amygdala and claustrum. In more posterior slices, the lateral ventricle composed the ventrolateral border and the hippocampus/alveus composed the ventromedial border. The region of white matter that includes the optic tract composed the dorsal border of the amygdala.
  The hippocampi were segmented primarily in the coronal and sagittal planes, similar to (50). In the sagittal plane, the lateral most slice was identified as where gray matter appeared in the temporal horn of the lateral ventricle. In the lateral slices, white matter defined the ventral border, and cerebrospinal fluid (CSF) defined the anterior and posterior borders. The dorsal border of the hippocampus was defined by two white matter structures, the alveus and fimbria. The alveus sits above the anterior portion of the hippocampus and was included in the segmentation. The fimbria is posterior to the alveus and was not included in the segmentation. In the medial slices, the curvature of the hippocampus causes it to appear in two sections, one anterior to the other. In both sections, white matter defined the ventral border. Also, a combination of white matter and CSF from the lateral ventricle defined the dorsal border. In the anterior section, the medial most slice was where the alveus converged with the white matter inferior to the hippocampus. In the posterior section, the medial-most slice is where the splenium of the corpus callosum appears.
  These guidelines include CA1, CA2, and CA3 regions of the hippocampus but exclude the subiculum.
4. The 5 atlas subjects were downsampled to 1 × 1 × 1 *mm*^3^ voxel size and then passed to MRICloud for *single-atlas* segmentation of coarse regions (“7 Label” Segmentation) such as gray matter, white matter, ventricles, CSF, skull, and background (51). The MRICloud atlas used was Adult22_50yrs_283Labels_26atlases_M2_V9B.
5. The automatic labels from Step 4 were upsampled to the original resolution then combined with the manual labels of hippocampi and amygdalae from Step 3.
6. Using the labeled atlas images from step 5, the LDDMM algorithm from MRICloud was used to perform automatic *multi-atlas* segmentation in the remaining 30 subjects to segment the hippocampi and amygdalae (45, 52, 53). Atlas information was based on segmentations of the atlas subjects from steps 3 and 4.
7. The 30 amygdala and hippocampus segmentations from Step 6 were reviewed and manually revised when necessary.

### 2.4. Shape Analysis via Surface-Based Morphometry

**Figure 1:**
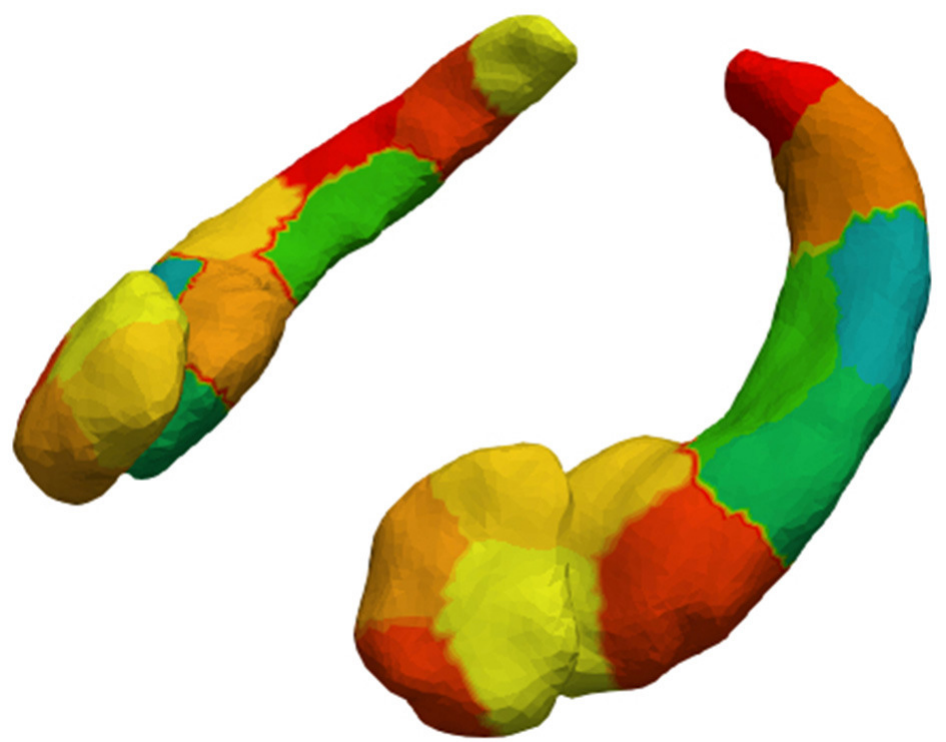
Amygdalar and hippocampal surface discretization from spectral clustering. After clustering, left and right amygdala are divided into 4 patches each and left and right hippocampus into 9 patches each.

Earlier works have described this method in more detail (35, 36, 54). Briefly,

1. Segmentations of the four structures (left/right amygdala and hippocampus) were converted to triangulated surfaces with Restricted Delaunay Triangulation (55).
2. The triangulated surfaces were passed to MRICloud to create population surface templates for both amygdalae and hippocampi (56). These templates serve as a common coordinate system for each subcortical structure.
3. The surface templates from step 2, and the triangulated surfaces from step 1 were passed to MRICloud to calculate deformations from each patient to the surface templates. The features on which this paper focuses are the surface Jacobians of the deformation at each vertex of the surface. The surface Jacobian measures the local expansion/atrophy around a particular vertex (56).
4. We downsampled the vertices into surface patches for computational efficiency. The surface patches were constructed with a spectral clustering method, which only relies on surface geometry (36). This method computes the first k eigenvectors of the Laplace-Beltrami operator associated with the surface. Then, each vertex is transformed into a k dimensional vector according to the corresponding elements in the eigenvectors. Finally, we cluster the vertices using the k-means algorithm. We downsampled the structures so the patches would have an average surface area of 150 *mm*^2^ (57). Figure 1 shows the 4 patches on the amygdalae, and the 9 patches on the hippocampi. The surface Jacobians of all vertices in a patch were averaged to obtain the local expansion/atrophy for that patch.

### 2.5. Statistical Analysis

Differences in demographic and clinical characteristics and volumes between the HC, responder, and non-responder groups were examined using chi-squared tests, one-way ANOVAs and two-sample *t*-tests implemented in MATLAB. We used general linear models to test for associations between groups (e.g., responder vs. non-responder) and brain shape. The method has been described in detail elsewhere (36). The same method was applied to each of the four structures being investigated, left and right amygdalae, and left and right hippocampi. After surface mapping described above, each participant had an expansion factor for each surface patch in the triangulated surfaces. The expansion factors associated with a brain structure were concatenated into a vector indexed by participant *i*: *y*_*i*_ (e.g., 4 dimensional vector for the left amygdala). These vectors describe how the brain structure of each participant differs from the template, or “average,” brain structure.

To determine whether clinical response status had a significant association with brain shape, we constructed a null linear model and alternative linear model. The alternative model included response status and the null model did not. Both models included covariates for sex, age, and intracranial volume (ICV). The model coefficients β were fit to minimize the sum of squared errors (across all subjects) between the predicted expansion factors and the actual expansion factors.

$$\begin{array}{l} Y=\left[\begin{array}{cccc} | & | & ... & |\\ y_{1} & y_{2} & ... & y_{n}\\ | & | & ... & | \end{array}\right]\;X=\left[\begin{array}{cccc} 1 & 1 & ... & 1\\ x_{1,age} & x_{2,age} & ... & x_{n,age}\\ x_{1,sex} & x_{2,sex} & ... & x_{n,sex}\\ x_{1,icv} & x_{2,icv} & ... & x_{n,icv}\\ x_{1,response} & x_{2,response} & ... & x_{n,response} \end{array}\right]\\ \;Y_{null}=\left[\begin{array}{ccccc} | & | & | & | & |\\ \beta_{intercept} & \beta_{age} & \beta_{sex} & \beta_{icv} & 0\\ | & | & | & | & | \end{array}\right]\;X\\ \;Y_{alt}=\left[\begin{array}{ccccc} | & | & | & | & |\\ \beta_{intercept} & \beta_{age} & \beta_{sex} & \beta_{icv} & \beta_{response}\\ | & | & | & | & | \end{array}\right]\;X \end{array}$$

In words, *Y*(*a, b*) corresponded to the expansion factor of the *a*th patch in participant *b* and *X*(*c, d*) corresponded to the *c*th covariate in participant *d*.

For each patch, the sum of squared errors across all subjects was computed for both models and the test statistic considered for patch *p* was $s_{p}=\frac{\overset{n}{\underset{i=1}{\sum }}{\left(Y\left(p,i\right)-Y_{null}\left(p,i\right)\right)}^{2}}{\overset{n}{\underset{i=1}{\sum }}{\left(Y\left(p,i\right)-Y_{alt}\left(p,i\right)\right)}^{2}}$. If the error at a patch was significantly lower in the alternative model, then the test statistic was large. A large test statistic implied that the feature was informative at that patch, i.e., the feature was associated with expansion or atrophy at that location. We used permutation testing to control the familywise error rate to 5% (58). A permutation test rearranged the features among the subjects and at each rearrangement, the maximum test statistic (across all surface patches) was used to form a permutation distribution. Then, the test statistics from the original, true feature arrangement, were compared to this permutation distribution. Any test statistic above the 95th percentile of the permutation distribution was considered significant.

## 3. Results

### 3.1. Demographic and Clinical Characteristics

Table 1 describes the distributions of demographic and clinical variables in each acute response group and in HC. As expected, participants with BD had higher levels of depressive symptoms than HC, with non-responders displaying the highest levels of depressive symptoms. Also expected, duration of treatment with lithium was longer for participants responding to lithium than those not responding. We did not detect significant between group differences for the other demographic or clinical variables tested.

**Table 1:** Demographic and clinical characteristics and brain volumes, by group.

	**Healthy comparison (*N* = 21)**	**Acute responder (*N* = 9)**	**Acute non-responder (*N* = 5)**	***p*-value**
				
**Demographic characteristics**				
Age (yrs)	36.3 (13.0)	37.7 (15.2)	31.0 (9.8)	0.65
Sex (% female)	71%	78%	100%	0.39
Education (yrs)	15.4 (2.9)	15.0 (2.5)	17.2 (1.8)	0.32
**Clinical characteristics**				
BDI	1.4 (1.9)	6.4 (6.5)	11.6 (7.3)	<0.01^*^
CARS-M	0.5 (1.3)	1.3 (2.4)	1.0 (2.2)	0.51
HART-FSIQ	110.4 (8.7)	124.9 (8.3)	123.9 (7.8)	<0.01^*^
**Volumes (*cm*^3^)**				
Intracranial (ICV)	1,503 (132)	1,472 (134)	1,406 (34)	0.30
**Volumes, normalized by ICV**				
Amygdala, left	0.65 (0.13)	0.69 (0.15)	0.66 (0.23)	0.80
Amygdala, right	0.69 (0.13)	0.71 (0.12)	0.70 (0.19)	0.93
Hippocampus, left	1.81 (0.22)	1.85 (0.20)	1.71 (0.25)	0.52
Hippocampus, right	1.61 (0.21)	1.68 (0.28)	1.61 (0.21)	0.73

*Shown are mean (standard deviation) unless otherwise indicated. Group differences tested using chi-square tests for dichotomous variables and F-tests for continuous variables. Post-hoc pairwise t-tests performed when evidence of a significant (p <0.05) group difference*.

* *For BDI and HART-FSIQ, p < 0.01 for post-hoc groupwise comparisons of responders vs. healthy comparison and non-responders vs. healthy comparison*.

### 3.2. Volume Results

Average ICV and ICV-normalized region of interest (ROI) volumes, are presented in Table 1. We did not detect significant between-group differences of ICV or normalized ROI volumes.

### 3.3. Shape Analysis

**Figure 2:**
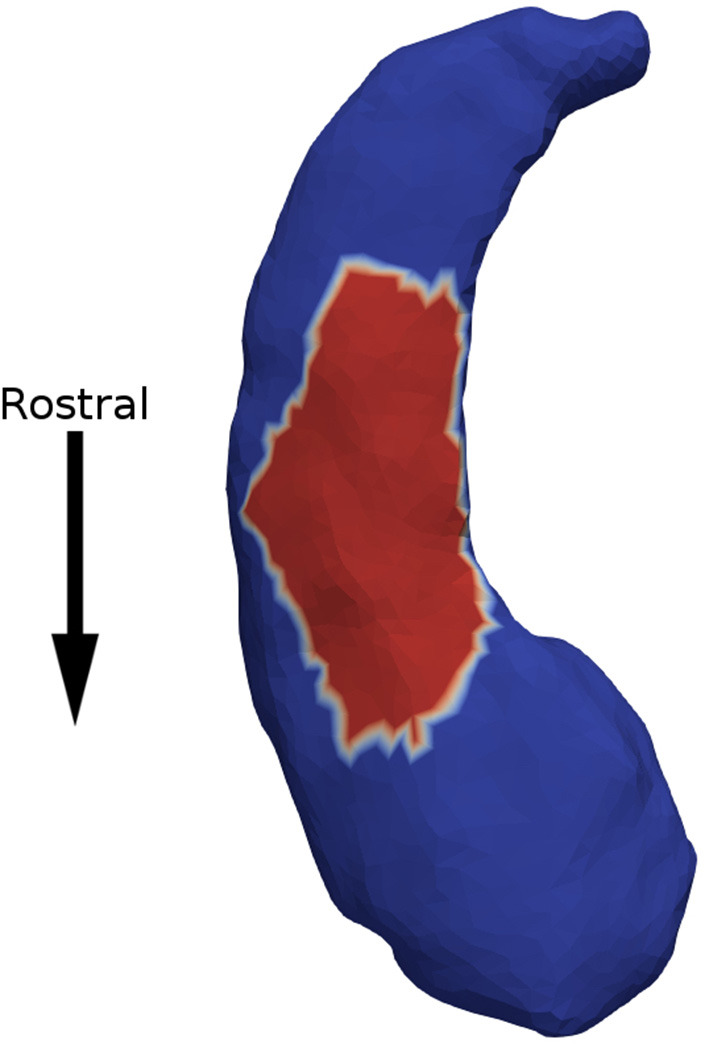
Non-responders were found to have 15% atrophy in a surface patch on the ventral side of the left hippocampus (in red), when compared to responders.

Minimum p-values across all patches are presented in Table 2. We observed a significant association between expansion factor and response status in the left hippocampus at the acute time point. Figure 2 shows that the patch significantly associated with response status is on the ventral surface, near the CA1, subiculum junction. This patch was atrophied by about 15% in non-responders when compared to the other groups. The association between expansion factor for this patch and response status maintained trend-level significance at the longer-term treatment time point for the comparison of non-responders vs. responders. We did not detect a significant association between expansion factor and response status in any other structure or patch.

**Table 2:** Associations between response status and expansion factor, minimum *p*-values.

		**Minimum patch *p*-value**	
		Acute	Long
Non-responders vs. Responders	Right amygdala	0.20	0.09
	Left Amygdala	0.61	0.90
	Right hippocampus	0.11	0.28
	Left hippocampus	0.03^*^	0.10
Non-responders vs. Healthy Comparison ∪ Responders	Right amygdala	0.11	0.13
	Left amygdala	0.51	0.76
	Right hippocampus	0.27	0.82
	Left hippocampus	0.03^*^	0.49

*Shown are the minimum p-value in each structure (minimum of 4 patches in amygdala or 9 patches in hippocampus). The first comparison is between non-responders and responders, the second comparison is between non-responders, and the combined group of healthy subjects and responders*.

* *For left hippocampus, minimum patch p < 0.05*.

## 4. Discussion

Results of this study provide evidence of lateralized morphometric differences in hippocampus in a group of patients with BD who did and did not respond to lithium treatment. We observed a significant difference in a region of the ventral left hippocampus, near the CA1/subiculum junction, which was relatively atrophied in non-responders as compared to responders. We did not find a significant difference in volumes of amygdalae or hippocampi between the lithium responders, non-responders, and HC groups. In this study, we examined individuals' responses to lithium treatment and localized differences in structure, factors that could help explain disparate findings related to effects of lithium use on structure of amygdala and hippocampus in the literature (17, 21, 31, 59, 60). In our sample, all participants with BD were treated with lithium monotherapy and were prospectively assessed for their response. Our surface mapping tools allowed for analysis of more localized shape changes that might not be detected by less detailed metrics like volume.

While identification of neuroimaging markers holds potential to predict treatment response, only a few previous studies have used neuroimaging to examine response to lithium treatment in BD. Task-based brain activation changes have been reported in lithium responders as compared to non-responders using functional MRI. One study found greater activation to an emotional faces task in prefrontal cortex and lesser activation in limbic regions in lithium responders as compared to non-responders (25). Another study comparing patients with first episode mania responding vs. not responding to either lithium or quetiapine observed differential changes in activation in subcortical regions in response to a continuous performance task with emotional distractors (29). Studies using functional MRI methods have also identified correlations between lithium response and amygdala-ventromedial prefrontal cortex functional connectivity and a normalizing effect of lithium on resting state connectivity measures (27, 28). Emerging research suggests these normalizing changes could come from neuroprotective effects of lithium against glutamatergic excitotoxicity or its association with higher levels of brain-derived neurotrophic factor (61). One study using structural MRI found a correlation between overall gray matter hypertrophy and clinical response to lithium in BD, but this study did not examine localized brain changes (26).

Most studies examining effects of lithium use on structural MRI have examined brain volumes, cortical thickness or surface area, reducing all morphological information to a single statistic (17, 21, 22, 30, 62). These studies have identified larger volumes of amygdalae and hippocampi in lithium-treated patients compared to patients not treated with lithium, although not consistently. In a large meta-analysis conducted by the ENIGMA consortium of 1,710 subjects with BD and 2,594 HC, though smaller hippocampi and amygdalae were observed in subjects with BD than in HC subjects, an effect of lithium use on these volumes was not found (17). Exploring more local effects in subcortical structure may provide additional information and potentially help further elucidate lithium's neurobiological action. A few studies have examined such structure at a more detailed level, testing volumes of hippocampal subfields, hippocampal thickness and subcortical shape (23, 31–34). These studies reported more localized differences in hippocampus including in CA1, CA2/3 and subiculum, although not consistently. However, these studies assessed structural changes related to lithium use and did not take into account differences in individual responses to lithium treatment.

We combined an examination of local effects in subcortical structure with a focus on individual differences in response to lithium treatment and observed a significant difference in a region of the ventral left hippocampus, near the CA1/subiculum junction, which was relatively atrophied in non-responders as compared to responders. CA1 is the primary output of the hippocampus and is integral in encoding memory related to space (63), novel objects (64), and fear (65). While there is limited literature on morphological differences between patients with BD taking and not taking lithium, our results are consistent with one previous study that identified smaller left CA1 and CA2/3 volumes in patients with BD not using lithium treatment than in a group using lithium, but only among participants with numerous affective episodes (33). Alterations in right hippocampus have also been reported, including a deficit in right CA1 in unmedicated patients with BD as compared to lithium treated patients and reduced volume of right CA2/3 and CA4/DG in patients with psychotic BD not taking vs. taking lithium (31, 32). Other studies have not detected a difference between lithium treated vs. not-treated patients with BD when examining measures of hippocampal shape (23, 34). We note that these inconsistencies in the literature could be at least partially explained by the focus on lithium treatment, not taking into account individual differences in response.

Our observation of a morphological difference in left hippocampus in lithium non-responders as compared to responders builds upon previous work describing lithium's effects in the brain. Using ^7^Li magnetic resonance imaging, euthymic patients with BD who were treated with lithium for 2 or more years were found to have the highest brain lithium content within a defined cluster in the left hippocampus (66). Additional support for a laterality effect in lithium response comes from a longitudinal study showing a decrease in left hippocampus volume over the course of treatment in patients with BD who were non-responders (67). Hippocampal laterality effects have also been shown with respect to patients with BD taking vs. not taking lithium, where left hippocampal volume or subfield volume has been shown to be smaller in those not taking lithium compared to those taking lithium or HC (33, 68, 69). Taken together, these findings suggest that left hippocampus may play a key role in lithium's mood stabilizing effects, and coupled with existing evidence of neurogenesis within the hippocampus lend support for the hypothesis of a neurogenic mechanism of action for lithium (70).

Interpretation of this study is limited by the small sample size. There are no males in our non-responder group, which may impact on the generalizability of our findings. There may exist potential confounding by clinical variables such as duration of illness (71), duration of treatment (21), depressive predominant polarity (72), or stressful life events (73) and these variables should be examined in a larger sample powered to do so. It is also important to note that the images in this study were collected after treatment was initiated so these results indicate correlations between brain shape and response, not predictors of response. Although we utilized a manual segmentation process, it was primarily performed by a single trained person blinded to clinical features and so should not differ systematically between groups. Subregions in this study were split along the surfaces and thus any changes occurring within the amygdalae or hippocampi would not have been detected. However, these methods could support deeper subregion analysis in future studies by segmenting images for each subregion, rather than for the whole amygdalae and hippocampi.

This study, to our knowledge, is the first *in-vivo* shape analysis of human brain structures in BD using 7T MRI. Previous morphological studies in humans used MRI field strengths of 3T or less (17, 21–23). Higher field strengths produce images with a higher signal to noise ratio (74) and might detect more subtle differences in neuroanatomy. MRICloud's implementation of LDDMM allowed for both a fast segmentation process and detection of localized shape changes in brain structures.

In order to answer the important question of how to predict lithium response in BD, larger and longitudinal neuroimaging studies are needed to establish whether there are any appreciable differences between responders and non-responders and whether those differences can predict response prior to treatment initiation or at an early stage of treatment. In this paper, we describe a possible approach to studying lithium response via neuroanatomy and report on a specific sub-region of the hippocampus, CA1, which may be associated with lithium response.

## Data Availability Statement

The datasets presented in this article are not readily available because they require specialized training to interpret. Requests to access the datasets should be directed to mood@bwh.harvard.edu.

## Ethics Statement

The studies involving human participants were reviewed and approved by Johns Hopkins Institutional Review Board. The patients/participants provided their written informed consent to participate in this study.

## Author Contributions

PBM, PPZ, JTR, JRD, JRK, and CMN contributed to the study design and implementation. TLA, CC, DJT, KSK, and PBM performed the data analysis. FSG, FM, KR-M, KG, and PBM acquired participant data. All authors contributed to the manuscript preparation.

## Conflict of Interest

JRD reports that he is Chairperson of the Board of Directors of the National Network of Depression Centers and receives reimbursement for official travel (amounting to less than $1500 annually). He has been an unpaid consultant for Myriad Neuroscience (formerly Assurex Health, Inc.) on behalf of the NNDC for meetings in 2017 and 2019. The NNDC was compensated for his effort. JRD owns stock in CVS-Health (275 shares valued today at just over $20,000). The remaining authors declare that the research was conducted in the absence of any commercial or financial relationships that could be construed as a potential conflict of interest.

## References

[1] Merikangas KR Akiskal HS Angst J Greenberg PE Hirschfeld RMA Petukhova M. Lifetime and 12-month prevalence of bipolar spectrum disorder in the National Comorbidity Survey replication. Arch Gen Psychiatry. (2007) 64:543–52. 10.1001/archpsyc.64.5.543 17485606 PMC1931566

[2] Kendall T Morriss R Mayo-Wilson E Marcus E. Assessment and management of bipolar disorder: summary of updated NICE guidance. Brit Med J. (2014) 349:g5673. 10.1136/bmj.g5673 25258392

[3] Wyatt RJ Henter ID Jamison JC. Lithium revisited: savings brought about by the use of lithium, 1970-1991. Psychiatr Q. (2001) 72:149–66. 10.1023/A:1010319610021 11433880 PMC4302724

[4] Geddes JR Burgess S Hawton K Jamison K Goodwin GM. Long-term lithium therapy for bipolar disorder: systematic review and meta-analysis of randomized controlled trials. Am J Psychiatry. (2004) 161:217–22. 10.1176/appi.ajp.161.2.217 14754766

[5] Yildiz A Vieta E Leucht S Baldessarini RJ. Efficacy of antimanic treatments: meta-analysis of randomized, controlled trials. Neuropsychopharmacology. (2011) 36:375. 10.1038/npp.2010.192 20980991 PMC3055677

[6] Rybakowski JK. Response to lithium in bipolar disorder: clinical and genetic findings. ACS Chem Neurosci. (2014) 5:413–21. 10.1021/cn5000277 24625017 PMC4063501

[7] Tighe SK Mahon PB Potash JB. Predictors of lithium response in bipolar disorder. Therapeut Adv Chronic Dis. (2011) 2:209–26. 10.1177/2040622311399173 PMC3513882 23251751

[8] Kleindienst N Engel RR Greil W. Which clinical factors predict response to prophylactic lithium? A systematic review for bipolar disorders. Bipolar Disord. (2005) 7:404–17. 10.1111/j.1399-5618.2005.00244.x 16176433

[9] Etain B Lajnef M Brichant-Petitjean C Geoffroy P Henry C Gard S. Childhood trauma and mixed episodes are associated with poor response to lithium in bipolar disorders. Acta Psychiatr Scand. (2017) 135:319–27. 10.1111/acps.12684 27987204

[10] Chen CH Lee CS Lee MTM Ouyang WC Chen CC Chong MY. Variant GADL1 and response to lithium therapy in bipolar I disorder. N Engl J Med. (2014) 370:119–28. 10.1056/NEJMoa1212444 24369049

[11] Hou L Heilbronner U Degenhardt F Adli M Akiyama K Akula N. Genetic variants associated with response to lithium treatment in bipolar disorder: a genome-wide association study. Lancet. (2016) 387:1085–93. 10.1016/S0140-6736(16)00143-4 26806518 PMC4814312

[12] Song J Bergen SE Di Florio A Karlsson R Charney A Ruderfer DM. Genome-wide association study identifies SESTD1 as a novel risk gene for lithium-responsive bipolar disorder. Mol Psychiatry. (2016) 21:1290–7. 10.1038/mp.2015.165 26503763 PMC4995544

[13] Perlis RH Smoller JW Ferreira MAR McQuillin A Bass N Lawrence J. A genomewide association study of response to lithium for prevention of recurrence in bipolar disorder. Am J Psychiatry. (2009) 166:718–25. 10.1176/appi.ajp.2009.08111633 19448189 PMC3908470

[14] Reinbold CS Forstner AJ Hecker J Fullerton JM Hoffmann P Hou L. Analysis of the influence of microRNAs in lithium response in bipolar disorder. Front Psychiatry. (2018) 9:207. 10.3389/fpsyt.2018.00207 29904359 PMC5991073

[15] Arnone D Cavanagh J Gerber D Lawrie SM Ebmeier KP McIntosh AM. Magnetic resonance imaging studies in bipolar disorder and schizophrenia: meta-analysis. Brit J Psychiatry. (2009) 195:194–201. 10.1192/bjp.bp.108.059717 19721106

[16] McDonald C Zanelli J Rabe-Hesketh S Ellison-Wright I Sham P Kalidindi S. Meta-analysis of magnetic resonance imaging brain morphometry studies in bipolar disorder. Biol Psychiatry. (2004) 56:411–7. 10.1016/j.biopsych.2004.06.021 15364039

[17] Hibar DP Westlye LT van Erp TGM Rasmussen J Leonardo CD Faskowitz J. Subcortical volumetric abnormalities in bipolar disorder. Mol Psychiatry. (2016) 21:1710–6. 10.1038/mp.2015.227 26857596 PMC5116479

[18] Phillips M Ladouceur C Drevets W. Neural systems underlying voluntary and automatic emotion regulation: toward a neural model of bipolar disorder. Mol Psychiatry. (2008) 13:829. 10.1038/mp.2008.82 18574483 PMC2745893

[19] Phillips ML Swartz HA. A critical appraisal of neuroimaging studies of bipolar disorder: toward a new conceptualization of underlying neural circuitry and a road map for future research. Am J Psychiatry. (2014) 171:829–43. 10.1176/appi.ajp.2014.13081008 24626773 PMC4119497

[20] Langan C McDonald C. Neurobiological trait abnormalities in bipolar disorder. Mol Psychiatry. (2009) 14:833–46. 10.1038/mp.2009.39 19455151

[21] Sani G Simonetti A Janiri D Banaj N Ambrosi E De Rossi P. Association between duration of lithium exposure and hippocampus/amygdala volumes in type I bipolar disorder. J Affect Disord. (2018) 232:341–8. 10.1016/j.jad.2018.02.042 29510351

[22] Savitz J Nugent AC Bogers W Liu A Sills R Luckenbaugh DA. Amygdala volume in depressed patients with bipolar disorder assessed using high resolution 3T MRI: the impact of medication. Neuroimage. (2010) 49:2966–76. 10.1016/j.neuroimage.2009.11.025 19931399 PMC2818548

[23] van Erp TGM Thompson PM Kieseppä T Bearden CE Marino AC Hoftman GD. Hippocampal morphology in lithium and non-lithium-treated bipolar I disorder patients, non-bipolar co-twins, and control twins. Hum Brain Mapp. (2012) 33:501–10. 10.1002/hbm.21239 21455943 PMC4383766

[24] Anand A Nakamura K Spielberg JM Cha J Karne H Hu B. Integrative analysis of lithium treatment associated effects on brain structure and peripheral gene expression reveals novel molecular insights into mechanism of action. Transl Psychiatry. (2020) 10:1–10. 10.1038/s41398-020-0784-z 32251271 PMC7136209

[25] Rootes-Murdy K Glazer K Mondimore FM Goes FS Zandi PP Bakker A. A pilot fMRI study of lithium response in bipolar disorder. Psychiatry Res Neuroimaging. (2019) 286:1. 10.1016/j.pscychresns.2019.02.003 30822677 PMC6749831

[26] Lyoo IK Dager SR Kim JE Yoon SJ Friedman SD Dunner DL. Lithium-induced gray matter volume increase as a neural correlate of treatment response in bipolar disorder: a longitudinal brain imaging study. Neuropsychopharmacology. (2010) 35:1743–50. 10.1038/npp.2010.41 20357761 PMC3055479

[27] Altinay M Karne H Anand A. Lithium monotherapy associated clinical improvement effects on amygdala-ventromedial prefrontal cortex resting state connectivity in bipolar disorder. J Affect Disord. (2018) 225:4–12. 10.1016/j.jad.2017.06.047 28772145 PMC5844774

[28] Spielberg JM Matyi MA Karne H Anand A. Lithium monotherapy associated longitudinal effects on resting state brain networks in clinical treatment of bipolar disorder. Bipolar Disord. (2019) 21:361–71. 10.1111/bdi.12718 30421491 PMC8593846

[29] Strakowski SM Fleck DE Welge J Eliassen JC Norris M Durling M. fMRI brain activation changes following treatment of a first bipolar manic episode. Bipolar Disord. (2016) 18:490–501. 10.1111/bdi.12426 27647671 PMC5951160

[30] Hibar DP Westlye LT Doan NT Jahanshad N Cheung JW Ching CRK. Cortical abnormalities in bipolar disorder: an MRI analysis of 6503 individuals from the ENIGMA bipolar disorder working group. Mol Psychiatry. (2018) 23:932–42. 10.1038/mp.2017.73 28461699 PMC5668195

[31] Bearden CE Thompson PM Dutton RA Frey BN Peluso MA Nicoletti M. Three-dimensional mapping of hippocampal anatomy in unmedicated and lithium-treated patients with bipolar disorder. Neuropsychopharmacology. (2008) 33:1229–38. 10.1038/sj.npp.1301507 17687266 PMC6693586

[32] Giakoumatos CI Nanda P Mathew IT Tandon N Shah J Bishop JR. Effects of lithium on cortical thickness and hippocampal subfield volumes in psychotic bipolar disorder. J Psychiatr Res. (2015) 61:180–7. 10.1016/j.jpsychires.2014.12.008 25563516 PMC4859940

[33] Hartberg CB JÃÿrgensen KN Haukvik UN Westlye LT Melle I Andreassen OA. Lithium treatment and hippocampal subfields and amygdala volumes in bipolar disorder. Bipolar Disord. (2015) 17:496–506. 10.1111/bdi.12295 25809287

[34] Vecchio D Piras F Piras F Banaj N Janiri D Simonetti A. Lithium treatment impacts nucleus accumbens shape in bipolar disorder. Neuroimage Clin. (2020) 25:102167. 10.1016/j.nicl.2020.102167 31972398 PMC6974785

[35] Miller MI Younes L Ratnanather JT Brown T Trinh H Lee DS. Amygdalar atrophy in symptomatic Alzheimer's disease based on diffeomorphometry: the BIOCARD cohort. Neurobiol Aging. (2015) 36:S3–10. 10.1016/j.neurobiolaging.2014.06.032 25444602 PMC4271320

[36] Younes L Ratnanather JT Brown T Aylward E Nopoulos P Johnson H. Regionally selective atrophy of subcortical structures in prodromal HD as revealed by statistical shape analysis. Hum Brain Mapp. (2014) 35:792–809. 10.1002/hbm.22214 23281100 PMC3715588

[37] Oedegaard KJ Alda M Anand A Andreassen OA Balaraman Y Berrettini WH. The Pharmacogenomics of Bipolar Disorder study (PGBD): identification of genes for lithium response in a prospective sample. BMC Psychiatry. (2016) 16:129. 10.1186/s12888-016-0732-x 27150464 PMC4857276

[38] Nurnberger JI Blehar MC Kaufmann CA York-Cooler C Simpson SG Harkavy-Friedman J. Diagnostic interview for genetic studies: rationale, unique features, and training. Arch Gen Psychiatry. (1994) 51:849–59. 10.1001/archpsyc.1994.03950110009002 7944874

[39] Beck AT Ward CH Mendelson M Mock J Erbaugh J. An inventory for measuring depression. Arch Gen Psychiatry. (1961) 4:561–71. 10.1001/archpsyc.1961.01710120031004 13688369

[40] Altman EG Hedeker DR Janicak PG Peterson JL Davis JM. The clinician-administered rating scale for mania (CARS-M): development, reliability, and validity. Biol Psychiatry. (1994) 36:124–34. 10.1016/0006-3223(94)91193-2 7948445

[41] Sheehan DV Lecrubier Y Sheehan KH Amorim P Janavs J Weiller E. The Mini-International Neuropsychiatric Interview (MINI): the development and validation of a structured diagnostic psychiatric interview for DSM-IV and ICD-10. J Clin Psychiatry. (1998) 59:22–33. 10.1037/t18597-000 9881538

[42] Schretlen DJ Winicki JM Meyer SM Testa SM Pearlson GD Gordon B. Development, psychometric properties, and validity of the Hopkins Adult Reading Test (HART). Clin Neuropsychol. (2009) 23:926–43. 10.1080/13854040802603684 19191072

[43] Folstein MF Folstein SE McHugh PR. “Mini-mental state”: a practical method for grading the cognitive state of patients for the clinician. J Psychiatr Res. (1975) 12:189–98. 10.1016/0022-3956(75)90026-6 1202204

[44] Nasreddine ZS Phillips NA Bédirian V Charbonneau S Whitehead V Collin I. The Montreal Cognitive Assessment, MoCA: a brief screening tool for mild cognitive impairment. J Am Geriatr Soc. (2005) 53:695–9. 10.1111/j.1532-5415.2005.53221.x 15817019

[45] Tang X Oishi K Faria AV Hillis AE Albert MS Mori S. Bayesian parameter estimation and segmentation in the multi-atlas random orbit model. PLoS ONE. (2013) 8:e65591. 10.1371/journal.pone.0065591 23824159 PMC3688886

[46] CIBC. Seg3D: Volumetric Image Segmentation and Visualization Scientific Computing and Imaging Institute (SCI) (2016). Available online at: http://www.seg3d.org

[47] Malone IB Leung KK Clegg S Barnes J Whitwell JL Ashburner J. Accurate automatic estimation of total intracranial volume: a nuisance variable with less nuisance. Neuroimage. (2015) 104:366–72. 10.1016/j.neuroimage.2014.09.034 25255942 PMC4265726

[48] Mai JK Majtanik M Paxinos G. Atlas of the Human Brain. Academic Press (2015).

[49] Entis JJ Doerga P Barrett LF Dickerson BC. A reliable protocol for the manual segmentation of the human amygdala and its subregions using ultra-high resolution MRI. Neuroimage. (2012) 60:1226–35. 10.1016/j.neuroimage.2011.12.073 22245260 PMC3665767

[50] Berron D Vieweg P Hochkeppler A Pluta JB Ding SL Maass A. A protocol for manual segmentation of medial temporal lobe subregions in 7 Tesla MRI. Neuroimage Clin. (2017) 15:466–82. 10.1016/j.nicl.2017.05.022 28652965 PMC5476466

[51] Ye C Ma T Wu D Ceritoglu C Miller MI Mori S. Atlas pre-selection strategies to enhance the efficiency and accuracy of multi-atlas brain segmentation tools. PLoS ONE. (2018) 13:e0200294. 10.1371/journal.pone.0200294 30052643 PMC6063392

[52] Mori S Wu D Ceritoglu C Li Y Kolasny A Vaillant MA. MRICloud: delivering high-throughput MRI neuroinformatics as cloud-based software as a service. Comput Sci Eng. (2016) 18:21–35. 10.1109/MCSE.2016.93

[53] Wang H Pouch A Takabe M Jackson B Gorman J Gorman R. Multi-atlas segmentation with robust label transfer and label fusion. In: International Conference on Information Processing in Medical Imaging. Springer (2013) p. 548–59. 10.1007/978-3-642-38868-2_46 PMC3974208 24683998

[54] Mahon PB Lee DS Trinh H Tward D Miller MI Younes L. Morphometry of the amygdala in schizophrenia and psychotic bipolar disorder. Schizophrenia Res. (2015) 164:199–202. 10.1016/j.schres.2015.02.011 25766598 PMC4439197

[55] Chew LP. Constrained Delauney tirangulations. Algorithmica. (1989) 4:97–108. 10.1007/BF01553881

[56] Ma J Miller MI Younes L. A Bayesian generative model for surface template estimation. Int J Biomed Imaging. (2010) 2010:1–14. 10.1155/2010/974957 20885934 PMC2946602

[57] Faria AV Ratnanather JT Tward DJ Lee DS van den Noort F Wu D. Linking white matter and deep gray matter alterations in premanifest Huntington disease. Neuroimage Clin. (2016) 11:450–60. 10.1016/j.nicl.2016.02.014 27104139 PMC4827723

[58] Nichols T Hayasaka S. Controlling the familywise error rate in functional neuroimaging: a comparative review. Stat Methods Med Res. (2003) 12:419–46. 10.1191/0962280203sm341ra 14599004

[59] Lopez-Jaramillo C Vargas C Diaz-Zuluaga AM Palacio JD Castrillon G Bearden C. Increased hippocampal, thalamus and amygdala volume in long—term lithium—treated bipolar I disorder patients compared with unmedicated patients and healthy subjects. Bipolar Disord. (2017) 19:41–9. 10.1111/bdi.12467 28239952

[60] Hajek T Kopecek M Hoschl C Alda M. Smaller hippocampal volumes in patients with bipolar disorder are masked by exposure to lithium: a meta-analysis. J Psychiatry Neurosci. (2012) 37:333. 10.1503/jpn.110143 22498078 PMC3447132

[61] Machado-Vieira R. Lithium, stress, and resilience in bipolar disorder: deciphering this key homeostatic synaptic plasticity regulator. J Affect Disord. (2018) 233:92–9. 10.1016/j.jad.2017.12.026 29310970

[62] Blumberg HP Kaufman J Martin A Whiteman R Zhang JH Gore JC. Amygdala and hippocampal volumes in adolescents and adults with bipolar disorder. Arch Gen Psychiatry. (2003) 60.12:1201–08. 10.1001/archpsyc.60.12.1201 14662552

[63] Hartley T Lever C Burgess N O'Keefe J. Space in the brain: how the hippocampal formation supports spatial cognition. Philos Trans R Soc B Biol Sci. (2014) 369:20120510. 10.1098/rstb.2012.0510 24366125 PMC3866435

[64] Cohen SJ Stackman RW Jr. Assessing rodent hippocampal involvement in the novel object recognition task. A review. Behav Brain Res. (2015) 285:105–17. 10.1016/j.bbr.2014.08.002 25169255 PMC7008635

[65] Izquierdo I Furini CR Myskiw JC. Fear memory. Physiol Rev. (2016) 96:695–750. 10.1152/physrev.00018.2015 26983799

[66] Stout J Hozer F Coste A Mauconduit F Djebrani-Oussedik N Sarrazin S. Accumulation of lithium in the hippocampus of patients with bipolar disorder: a lithium-7 magnetic resonance imaging study at 7 Tesla. Biol Psychiatry. (2020) 88:426–33. 10.1016/j.biopsych.2020.02.1181 32340717

[67] Selek S Nicoletti M Zunta-Soares GB Hatch JP Nery FG Matsuo K. A longitudinal study of fronto-limbic brain structures in patients with bipolar I disorder during lithium treatment. J Affect Disord. (2013) 150:629–33. 10.1016/j.jad.2013.04.020 23764385

[68] Hajek T Cullis J Novak T Kopecek M Höschl C Blagdon R. Hippocampal volumes in bipolar disorders: opposing effects of illness burden and lithium treatment. Bipolar Disord. (2012) 14:261–70. 10.1111/j.1399-5618.2012.01013.x 22548899 PMC3525647

[69] Zung S Souza-Duran F Soeiro-de Souza M Uchida R Bottino C Busatto G. The influence of lithium on hippocampal volume in elderly bipolar patients: a study using voxel-based morphometry. Transl Psychiatry. (2016) 6:e846. 10.1038/tp.2016.97 27351600 PMC4931614

[70] Toda T Gage FH. Adult neurogenesis contributes to hippocampal plasticity. Cell Tissue Res. (2018) 373:693–709. 10.1007/s00441-017-2735-4 29185071

[71] Javadapour A Malhi GS Ivanovski B Chen X Wen W Sachdev P. Hippocampal volumes in adults with bipolar disorder. J Neuropsychiatry Clin Neurosci. (2010) 22:55–62. 10.1176/jnp.2010.22.1.55 20160210

[72] Janiri D Simonetti A Piras F Ciullo V Spalletta G Sani G. Predominant polarity and hippocampal subfield volumes in Bipolar disorders. Bipolar Disord. (2020) 22:490–7. 10.1111/bdi.12857 31630469

[73] Janiri D Sani G De Rossi P Piras F Banaj N Ciullo V. Hippocampal subfield volumes and childhood trauma in bipolar disorders. J Affect Disord. (2019) 253:35–43. 10.1016/j.jad.2019.04.071 31022627

[74] Vaughan JT Garwood M Collins CM Liu W DelaBarre L Adriany G. 7T vs. 4T: RF power, homogeneity, and signal-to-noise comparison in head images. Magnet Reson Med. (2001) 46:24–30. 10.1002/mrm.1156 11443707

